# Leveraging big data for improving the estimation of close to reality travel time to obstetric emergency services in urban low- and middle-income settings

**DOI:** 10.3389/fpubh.2022.931401

**Published:** 2022-07-29

**Authors:** Aduragbemi Banke-Thomas, Peter M. Macharia, Prestige Tatenda Makanga, Lenka Beňová, Kerry L. M. Wong, Uchenna Gwacham-Anisiobi, Jia Wang, Tope Olubodun, Olakunmi Ogunyemi, Bosede B. Afolabi, Bassey Ebenso, Ibukun-Oluwa Omolade Abejirinde

**Affiliations:** ^1^School of Human Sciences, University of Greenwich, London, United Kingdom; ^2^Maternal and Reproductive Health Research Collective, Lagos, Nigeria; ^3^Population Health Unit, Kenya Medical Research Institute-Wellcome Trust Research Programme, Nairobi, Kenya; ^4^Centre for Health Informatics, Computing, and Statistics, Lancaster Medical School, Lancaster University, Lancaster, United Kingdom; ^5^Surveying and Geomatics Department, Faculty of Science and Technology, Midlands State University, Gweru, Zimbabwe; ^6^Department of Public Health, Institute of Tropical Medicine, Antwerp, Belgium; ^7^Department of Infectious Disease Epidemiology, London School of Hygiene and Tropical Medicine, London, United Kingdom; ^8^Nuffield Department of Population Health, University of Oxford, Oxford, United Kingdom; ^9^School of Computing and Mathematical Sciences, University of Greenwich, London, United Kingdom; ^10^Department of Community Medicine and Primary Care, Federal Medical Centre Abeokuta, Abeokuta, Nigeria; ^11^Lagos State Ministry of Health, Lagos, Nigeria; ^12^Department of Obstetrics and Gynaecology, College of Medicine, University of Lagos, Lagos, Nigeria; ^13^Leeds Institute of Health Sciences, University of Leeds, Leeds, United Kingdom; ^14^Women's College Hospital Institute for Health System Solutions and Virtual Care, Toronto, ON, Canada; ^15^Dalla Lana School of Public Health, University of Toronto, Toronto, ON, Canada

**Keywords:** urbanization and developing countries, emergency obstetric care, access, equity, travel time, big data, digital technology

## Abstract

Maternal and perinatal mortality remain huge challenges globally, particularly in low- and middle-income countries (LMICs) where >98% of these deaths occur. Emergency obstetric care (EmOC) provided by skilled health personnel is an evidence-based package of interventions effective in reducing these deaths associated with pregnancy and childbirth. Until recently, pregnant women residing in urban areas have been considered to have good access to care, including EmOC. However, emerging evidence shows that due to rapid urbanization, this so called “*urban advantage”* is shrinking and in some LMIC settings, it is almost non-existent. This poses a complex challenge for structuring an effective health service delivery system, which tend to have poor spatial planning especially in LMIC settings. To optimize access to EmOC and ultimately reduce preventable maternal deaths within the context of urbanization, it is imperative to accurately locate areas and population groups that are geographically marginalized. Underpinning such assessments is accurately estimating travel time to health facilities that provide EmOC. In this perspective, we discuss strengths and weaknesses of approaches commonly used to estimate travel times to EmOC in LMICs, broadly grouped as reported and modeled approaches, while contextualizing our discussion in urban areas. We then introduce the novel OnTIME project, which seeks to address some of the key limitations in these commonly used approaches by leveraging big data. The perspective concludes with a discussion on anticipated outcomes and potential policy applications of the OnTIME project.

## Introduction

Maternal mortality remains a huge challenge in many countries globally, with its burden substantially higher in low- and middle-income countries (LMICs) where 99% of maternal deaths occur (1). Despite a 38% reduction in global maternal mortality between 2000 and 2017, ~295,000 maternal deaths occur annually from preventable causes related to pregnancy and childbirth (1). Similarly, 98% of the three million perinatal deaths reported globally occurs in LMICs (2). These deaths are mostly associated with complications of pregnancy and childbirth, including pre-eclampsia/eclampsia, hemorrhage, sepsis, and abortion (3). Presently, the consensus strategy for minimizing pregnancy and childbirth related deaths is mainly focused on increasing access to prompt emergency obstetric care (EmOC) provided by skilled health personnel (4, 5). EmOC is a package of nine clinical and surgical evidence-based interventions including parenteral antibiotics, uterotonic drugs, parenteral anticonvulsants, manual removal of placenta, removal of retained products of conception, assisted vaginal delivery, neonatal resuscitation, blood transfusion and cesarean section (5). EmOC has been shown to reduce maternal deaths amongst women who reach health facilities by 15–50% and intrapartum stillbirths by 45–75% (6).

In emergency situations, pregnant women with obstetric complications need to travel to health facilities with capacity to provide EmOC. Delays in reaching such health facilities significantly affects pregnancy outcomes for mothers and newborns (7–10). Many of the health facilities that pregnant women with obstetric emergencies require for care are hospitals, classed as secondary and tertiary level health facilities, which are often located in urban settings. Women living in urban areas have been assumed to have better physical access EmOC compared to their rural counterparts due to relatively shorter travel distances to health facilities (11). However, emerging evidence shows that this so called “*urban advantage”* is shrinking and, in some LMIC settings, almost non-existent partly because while travel distances might be shorter, travel time can get longer (9, 12, 13). In urban LMIC settings, typically characterized by poor spatial planning, haphazardly built environments, growing informal settlements, poor road infrastructure, and extreme traffic congestion prolong travel time, delay care-seeking, and aggravate the risk of long-term morbidity and mortality for women and their babies. When these issues are considered against the backdrop of rapid urbanization in which 70% of the world's population is expected to live in urban areas by 2050, with nearly 90% of the projected additional 2.5 billion urban residents concentrated in Africa and Asia alone (14), urgent action is needed for service planning.

To optimize access to EmOC and ultimately reduce preventable maternal deaths within the context of urbanization, it is imperative to accurately identify areas and population groups that are physically marginalized in urban areas. Identification of these areas of geographical inequities will provide a useful starting point to engage in dialogues with policymakers on urban health and planning, as well as to inform multisectoral policies and action. Underpinning such assessments of geographical inequities are accurate estimates of travel time to EmOC facilities. In this perspective paper, we discuss strengths and weaknesses of the commonly used approaches in estimating travel time to EmOC services in LMICs, contextualizing our discussion in urban areas. We then introduce a novel initiative called the OnTIME project, which is attempting to address some of the key limitations in the commonly used approaches in LMICs by leveraging big data. The perspective concludes with a discussion on anticipated outcomes and potential policy applications of the project.

## Common methods for estimating travel time to EmOC in LMICs

Broadly, methods that have been used for estimating travel time to EmOC in LMICs can be grouped into reported and modeled approaches. Reported approaches entail asking health workers or women to estimate their travel times to health facilities. Some concerns with this approach have been raised. First, since health workers themselves did not make the journeys, their estimates are at best “guestimates” of the journeys that women might have undertaken to reach the health facility. In cases where women are asked to report their travel time, issues of recall bias have been highlighted by researchers, especially as they traveled in an emergency (15, 16).

**Figure 1:**
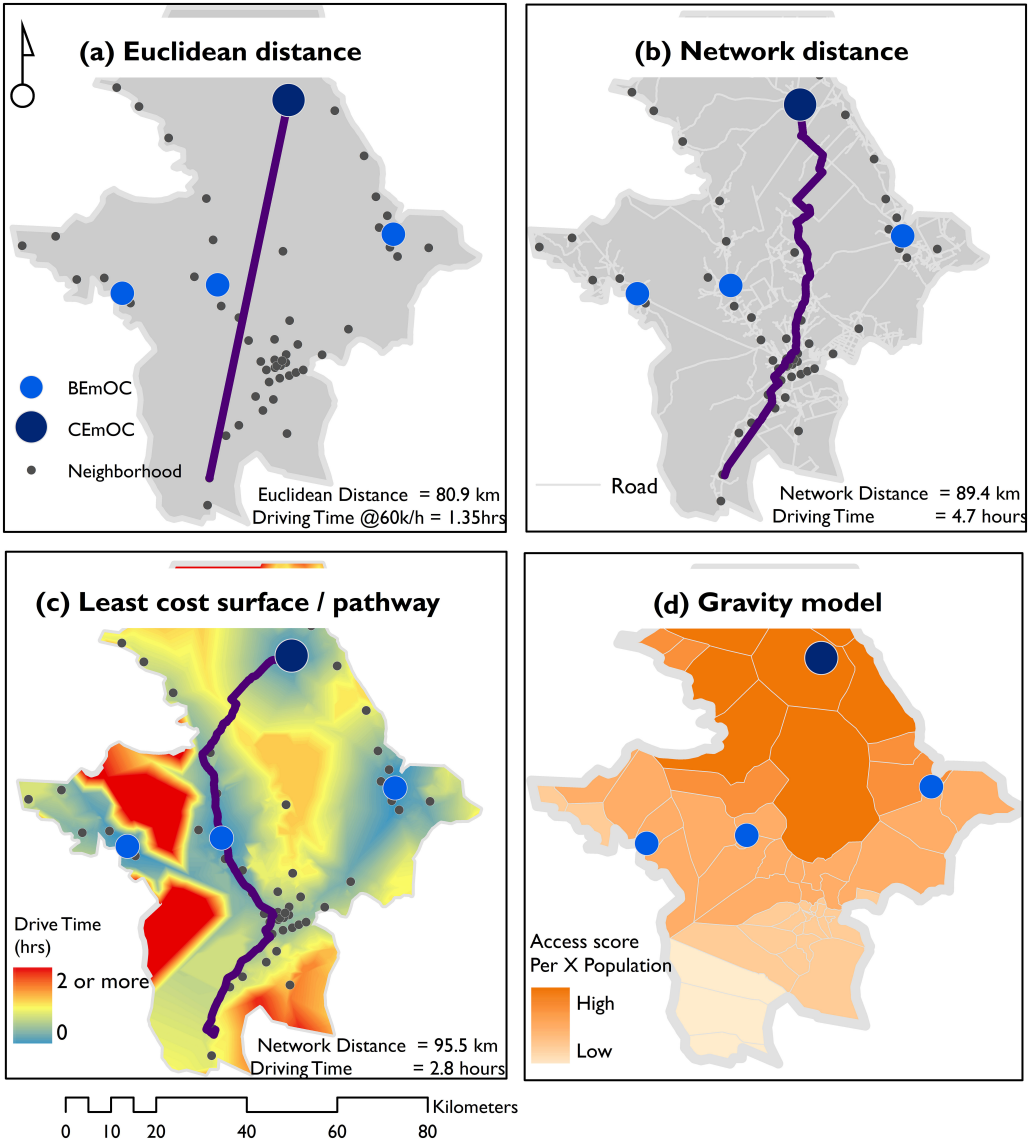
Common methods for estimating travel time to health facilities in LMICs. An illustration of common approaches used to compute geographic access to health service providers in LMICs including **(a)**, Euclidean distance, **(b)** network distance, **(c)** least cost path distance, and **(d)** gravity models.

On the other hand, modeled approaches are commonly used to estimate travel time to health facilities in LMICs (17). They range from simple approaches such as Euclidean model, to sophisticated methods that include network analysis, cost distance analysis and gravity models as summarized in Figure 1. These methods have been detailed by Ouma et al. (17). Briefly, Euclidean distances are the simplest to compute and assume straight line of travel from residence to EmOC locations, however, they ignore the influence of transport variables such as travel barriers, road network and slope. Gravity models combines availability and accessibility across defined spatial units to overcome this limitation of Euclidean approaches. However, the method may suffer from the modifiable areal unit problem, is dependent on the availability of population at very fine geographic units and service provider capacity data which are not always available in resource limited settings. Network analysis entails computing travel time along existing travel routes to a specified health facility. The method relies on a well-mapped transportation network and settlements, assumes travel can only occur along the roads and it is computationally intensive. On the other hand, cost distance analysis relies on travel speeds across land covers, road network and elevation, to define the least time needed to get to a health facility from residences. A common problem underlying all these approaches is reliance of empirical data (which is rarely available) to parametrise a model that represents the journey between where a need is triggered and the location of the service provider (18).

Despite the widespread application of modeled approaches, they have a range of known limitations, some of which are accentuated by the intrinsic dynamics and variability of conditions that typify urban contexts (19). One fundamental limitation is that it is hard to create accurate models that replicate actual journeys. This is often due to inadequate data on where the journey was initiated, the health facility visited, the route used, its condition at the time of travel (traffic, weather, accidents), the mode(s) of transport and the speed of travel. As a result, empirical models tend to make assumptions about travel speeds and mode of transport, rarely accounting for the dynamism of traffic conditions, weather conditions, and unforeseen travel circumstances such as waiting time, police checkpoints, or impassability of roads. As regards traffic in particular, urbanization and the expansion of the middle class in urban LMIC areas have resulted in a rapid increase in vehicular traffic, leading to significant traffic congestion (20). For example, commuters in Lagos, the largest megacity in sub-Saharan Africa spend an average of 30 h a week (equivalent to 75% of a 40-h working week) in traffic, with some taking up to 3 h to travel 10 km (21). In Asia, three megacities of India—Bengaluru, Mumbai, and New Delhi—are part of the top 10 highly ranked cities with populations over eight million and with the highest levels of traffic congestion across the globe (22). Previous research which compared modeled travel time estimates with those collected from replication of travel journeys made by pregnant women in Lagos showed that while the median replicated drive time was 50–52 min, mean errors of >45 min were reported for the cost-friction surface approach and Open Street Route Mapping (23). Ignoring variability in traffic conditions results in as much as three-fold overestimation of geographic coverage and masks intra-urban inequities in accessibility to emergency care (19). Another limitation of modeled approaches, as they have been commonly used, relates to establishing the travel destination. Majority of the modeled approaches estimate travel time to the nearest health facility. Yet, it is well established that even in emergencies, pregnant women may bypass the nearest health facility for a myriad of reasons including trust, cost, and the real or perceived quality of care. Women might also be referred from one facility to others. When this occurs, their journeys are typically a lot more complex, harder to model and does not always follow the path of the least resistance a common approach to modeled approaches (24–27). These limitations can result in underestimated time to access care, with significant implications for underserved populations that require targeted policies and action (28).

The constraints reported by researchers regarding pushing the frontier to reflect closer-to-reality travel time estimates relate to capacity to accurately parametrise a model that mimics the dynamics of the journey between the residence and service provider (29, 30). Data required for improved model parameterization include residential location of service users, location of the utilized facility providing EmOC, route used, mode of transport, traffic and weather variables, travel speed and transport barriers, among other travel dynamics (31). However, collecting such data is time-consuming, expensive, and probably impractical especially in low resource settings where there are many competing needs for resources. Also, the dynamics change dramatically, so data from last year or even last month may become less useful for understanding travel of mothers in an emergency. To move forward, such data needs to be real-time or at least close to real time.

## Leveraging big data for EmOC access

We have previously shown that Google Maps provides closer to reality estimates relative to modeled estimates based on travel time derived from replicated journeys of women seeking EmOC services (23). Consequently, we conducted a study using Google Maps to assess travel time of pregnant women to EmOC in Lagos, Nigeria—the most populated metropolis in sub-Saharan Africa (32). Our state-wide application of this method clearly showed areas of geographical inequity, one of which aligned with a gap in EmOC access that the Lagos state government addressed during the year of the study and others matched areas requiring attention to improve EmOC access (32, 33). Post-study dissemination efforts confirmed the high value that policymakers place on the insights generated using closer-to-reality estimates (34). Building on this success, the “On Tackling In-transit delays for Mothers in Emergency” (OnTIME) project is a novel initiative bringing together researchers, policy makers and the digital technology sector to leverage big data to generate closer-to-reality assessments of travel time to EmOC services in urban LMIC settings (www.ontimeconsortium.org/).

To achieve the project's goal, data on travel time and functionality of public and private hospitals in selected LMIC urban settings will be utilized. The travel time data will be computed using Google Maps Platform Directions API, the same one that helps calculate directions in Google Maps. Google Navigation uses real-time traffic conditions along with historical traffic patterns and road network data to predict accurate travel times. Therefore, the role of traffic congestion, time of the day, day of week, weather variation, and other unpredictable events will be indirectly embedded. Data on facility functionality will be collected from existing health facility registries in LMIC countries and verified by the OnTIME research team. Putting both datasets together will help characterize travel time to facilities providing EmOC. Furthermore, data on mode of transport commonly used by pregnant women in the country and in urban areas under consideration will be sourced to further refine travel time estimates. Finally, recognizing that obstetric referral patterns vary (35), the project will provide estimates accounting for different pathways in urban LMIC settings, including through referral. The plan is to then feed all the data into a digital dashboard with different visualization and scenario building options that can guide decisions around service delivery modalities, infrastructural and transportation priorities, and locations of future health facilities. It will also aid a more accurate estimation of the gaps between demand and supply of EmOC at a population level. Such evidence will be invaluable for service planning and policymaking, as closer-to-reality travel times reduce the number of generalizations and assumptions typically applied in empirical models in urban settings.

The pilot phase of the OnTIME project will focus on urban settings with an estimated population greater than one million in Nigeria. Outputs of this phase will be used to further refine the approach which could then be applied during subsequent project phases when the focus expands to other urban LMIC conurbations.

## Discussion

Urbanization poses a complex challenge for structuring an effective health service delivery system that is inclusive and responsive, especially in LMICs. It is an even greater challenge when consideration is given to the sprawling slum areas in many LMICs (36). Many of those living in slums are poor, who tend to live in unsafe conditions and have limited access to personal means of transport. This is driving widespread intra-urban inequities placing the urban poor at higher risks of poor outcomes even though health services are available and seemingly within reach (12, 37).

Clearly the roadmap toward equitable and responsive urban health service planning needs to rely on closer-to-reality travel time data that is available and valid—a task that the OnTIME Consortium is taking on, one urban LMIC setting at a time. The activities of the consortium are geared toward the development of a co-produced, context-specific, and action-oriented dashboard to support evidence-based decision-making and to guide targeted investments needed to support the advancement of robust urban health systems. Our expectation is that this dashboard and the underlying dataset will contribute to improving access to care and ultimately in reducing urban maternal and perinatal mortality in LMICs. The more accurate methods for estimating time to travel are, the better-informed urban planners and policy makers will become.

The OnTIME project has potential to inform decision-making for service planning on a granular, grassroot and closer-to-the-community basis is clear, as it will provide the evidence base needed for strategic response to EmOC service provision (38). To deal with issue of bypassing, the project also brings in the important element of choice that women have in deciding which hospital they go to in an emergency by assessing the first, second and third nearest public and private options available to the pregnant woman, since it is widely recognized that several non-travel-related factors influence the choice of where women seek EmOC (24, 39). Of course, a woman may still choose to go farther away from home for care in an emergency, but our approach of including options is a step change in the field of accessibility assessments. Our approach allows for prospective assessments which will be useful for service planning. As has already been established, our approach also allows for retrospective assessments when actual residence location and attribute data on the hospital that was utilized are available (32).

The promise of the OnTIME project in tackling in-transit delays for mothers in emergencies goes beyond its application for the supply-side focus. There is also a huge potential for the OnTIME approach to serve demand-side innovation which can inform health-seeking and choice of facility for EmOC amongst pregnant women. This will be very important as there is evidence that travel time strongly influence hospital choice, even in urban areas where alternatives are widely available (40). Despite the promise, some key gaps will still need to be addressed in future. Health care access is multi-dimensional and entails availability (physical availability), acceptability (perception of quality), accommodation (structures to support care access), affordability (cost), and accessibility (geographical accessibility) (41). While the precise nature of influences of acceptability, accommodation, affordability, and availability on EmOC access will need to be incorporated in future efforts, the OnTIME consortium is currently focused on accessibility. Capturing closer-to-reality data on EmOC accessibility constitutes the very next frontier for policy and research in EmOC access in urban LMIC settings, as the other access dimensions can only be properly understood if accessibility is reflective of reality. The OnTIME project will generate this evidence and in so doing, this initiative aims to advance the urban health agenda for equitable and responsive health systems and contribute to global efforts to reduce maternal and perinatal mortality (42).

## Data availability statement

The original contributions presented in the study are included in the article/supplementary material, further inquiries can be directed to the corresponding author.

## Author contributions

AB-T, PMM, PTM, LB, and IOA conceptualized the study and prepared the first draft of the manuscript. AB-T, PMM, PTM, LB, KW, UG-A, and IOA conducted the literature review that informed the study. AB-T, PMM, PTM, KW, UG-A, JW, and IOA synthesized the retrieved data. All authors were involved in the preparation of subsequent drafts and approved the final version.

## Funding

The OnTIME project led by AB-T was funded by Google. AB-T and BA were funded by Bill and Melinda Gates Foundation (Investment ID: INV-032911). PMM was supported by Newton International Fellowship (Number NIF/R1/201418) of the Royal Society and acknowledges the support of the Wellcome Trust to the Kenya Major Overseas Programme (Number 203077). LB was funded in part by the Research Foundation–Flanders (FWO) as part of her Senior Postdoctoral Fellowship.

## Conflict of interest

The authors declare that the research was conducted in the absence of any commercial or financial relationships that could be construed as a potential conflict of interest.

## Publisher's note

All claims expressed in this article are solely those of the authors and do not necessarily represent those of their affiliated organizations, or those of the publisher, the editors and the reviewers. Any product that may be evaluated in this article, or claim that may be made by its manufacturer, is not guaranteed or endorsed by the publisher.
